# Incidence rate of osteoporotic hip fracture in Qatar

**DOI:** 10.1007/s11657-021-01010-8

**Published:** 2021-10-05

**Authors:** Omar Suhail Alsaed, Nabeel Abdulla, Abdo Lutf, Ibrahim Abdulmomen, Fiaz Alam, Samar A. Razaq Alemadi

**Affiliations:** Division of Rheumatology, Department of Medicine, Hamad Medical Corporation, Alrayyan Street, PO BOX 3050, Doha, Qatar

**Keywords:** Osteoporosis, Hip fracture, Osteoporotic fracture, Age-sex-specific incidence rate, Age-adjusted standardized incidence rate

## Abstract

***Summary*:**

The incidence rate of osteoporotic hip fracture is essential to formulate a national fracture risk assessment tool (FRAX). In this epidemiological study, the incidence rate of osteoporotic hip fracture in Qatar was comparable to that in regional countries, and lower than that in North America and European countries.

**Purpose:**

Estimate the annual incidence rate (IR) of osteoporotic hip fractures (OHF) in Qatar from January 2017 to December 2019.

**Methods:**

Hamad Medical Corporation is a government-based tertiary medical institute. Hip fractures were captured by using the International Classification of Diseases-10 hip fracture codes. The patient records were reviewed retrospectively to identify fracture mechanisms. The observed census in 2017 and the estimated censuses of 2018 and 2019 were used to calculate the age-sex-specific annual IR of OHF in the population aged ≥ 40 years. The world population in 2010 was used to calculate the age-adjusted standardized IR in the population aged ≥ 50 years.

**Results:**

In total, 458 hip fractures were identified; 75 (16.4%) were due to high-energy trauma, and 9 (2%) were pathological hip fractures. The total number of OHF was 374 (81.7%). OHF was slightly higher in men (215, 57.5%). The median age (IQR) of the patients was 69 years (56–78 years). In 2017, 2018, and 2019, the age-adjusted standardized IR of OHF per 100,000 with the corresponding 95% CI was 141.7 (141.1–142.2), 140.8 (140.2–141.3), and 162.7 (162.0–163.2) for the whole Qatar population; 154.2 (153.6–154.7), 105.2 (104.7–105.7), and 176.6 (175.9–177.1) for Qataris; and 134.8 (134.3–135.4), 183.9 (183.3–184.6), and 160.4 (159.8–161.0) for non-Qataris, respectively.

**Conclusion:**

The annual age-adjusted standardized IR of OHF per 100,000 inhabitants aged ≥ 50 years in Qatar was comparable to that in regional countries, and lower than that in North America and European countries.

## Introduction

Osteoporosis is a global health problem, which is associated with fragility fractures of the spine, hip, humerus, and wrist. Hip fracture is a devastating incident that carries a high mortality rate, negatively affects the quality of life of survivors, and burdens the economy of the health care system [1, 2]. Following a hip fracture, 40 to 60% of patients recover their pre-fracture mobility status, 40–70% regain their level of independence for basic activities of daily living, and 10–20% are institutionalized following fracture [3]. It is estimated that the incidence of hip fracture is projected to increase by 240% in women and 310% in men by 2050 [4]. The Asian Federation of Osteoporosis Societies study showed that the incidence of hip fracture is expected to increase by 2.28-fold in 2050 [5].

The incidence rate of osteoporotic hip fracture is one of the essential indices used to predict the probability of fragility fractures and can be used to formulate a national fracture risk assessment tool (FRAX). Most osteoporosis society recommendations favor the use of the FRAX tool to determine the threshold for commencing osteoporotic treatment for each population [6]. There is a large variation in the incidence of hip fractures among different countries and regions worldwide [7]. Based on the available published reports, the incidence is highest in Scandinavian and European countries (> 350 per 100,000) [8, 9] and lowest in African countries (< 100 per 100,000) [10–13]. More hip fracture data are being published in the Middle East and Arab countries. Accumulating data over several decades is essential to provide a broad insight into the probability of fragility fracture rather than simply having data from one country at one point in time.

The structure and number of the Qatar population have changed dramatically over the last 20 years. In 2005, the Qatar population was around 668,629, while in 2017, the Qatar population was 2.7 million, the majority of whom were expatriates from different countries. This figure was obtained from “Qatar Population and Employment Projections 2017–2024—a framework for national planning.”

The aim of this study was to estimate the incidence rate of osteoporotic hip fractures in the Qatar population from January 2017 to December 2019. We believe that these data will help policymakers to formulate future plans for osteoporosis management and fragility fracture prevention.

## Materials and methods

### Study settings

Hamad Medical Corporation (HMC) is a government-based non-profit medical institute that provides medical care to all people in different municipalities of Qatar. The electronic medical records (EMRs) of all hospitals operated by HMC (Hamad General Hospital, Al Wakra Hospital, and Al Khor Hospital) were electronically linked by Cerner®. Each citizen and non-citizen resident in Qatar has a unique health care number. The present study included all adult patients aged ≥ 40 years who presented to the outpatient clinics and emergency room, or were admitted with hip fracture between January 2017 and December 2019.

### Case identification

The International Classification of Diseases (ICD-10) was used for disease coding in all hospitals operated by HMC. Hip fractures were captured by identifying ICD-10 hip fracture codes (S72.0, S72.1, and S72.2) for individuals aged ≥ 40 years from January 2017 to the end of December 2019. The EMR of these patients was retrospectively reviewed to identify patient demographic data, and the incidence of low-energy trauma hip fractures, high-energy trauma hip fractures, and pathological hip fractures. The standard definition of a low-trauma hip fracture was used to define osteoporotic hip fracture. Low-energy trauma hip fracture was defined as trauma arising from falling from standing height, or trauma, which, in a healthy individual, would not have resulted in a fracture. Pathological fractures were not considered osteoporotic fractures. Patients who were visitors to Qatar when they developed hip fracture were excluded.

### Incidence rates and standardized incidence rates

**Fig. 1:**
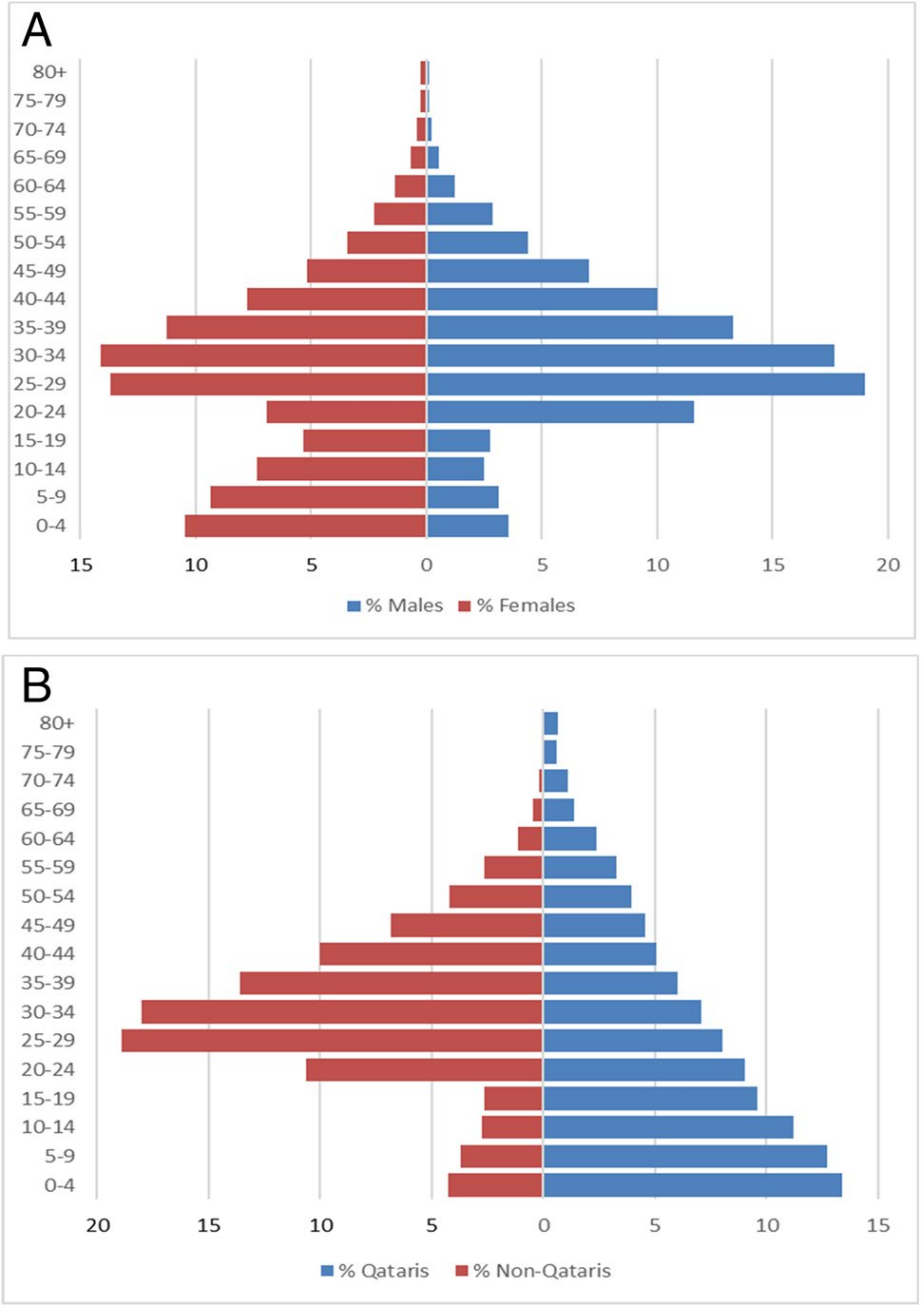
**A** Qatar population age pyramid based on the 2017 observed census. **B** Qatar population distributed according to Qataris and non-Qataris based on the 2017 observed census

Age-sex-specific incidence rates for 2017–2019 were defined as the yearly number of new osteoporotic hip fracture events divided by the number of individuals from the same sex and age group. To identify the possible impact of age on the observed trends in incidence rates over time, age groups were classified at 5-year intervals starting from 40 years and ending with 80 years and above (80 +). The population census provided by the book “Qatar Population and Employment Projections 2017–2024—a framework for national planning” was used to calculate the annual age-sex-specific incidence rate of osteoporotic hip fracture. The 2017 census involved observed data, while the 2018 and 2019 censuses comprised estimated data, according to the projection mentioned in the framework. Figure 1A shows Qatar population age pyramid based on the 2017 observed census. Figure 1B shows Qatar population distributed according to Qataris and non-Qataris based on the 2017 observed census. To enable comparisons with other populations, the incidence rates were standardized to the world population of 2010, using the numbers reported by the United Nations (UN) (esa.un.org/unpd/wpp/). The age-adjusted standardized incidence rate with the corresponding 95% confidence interval (CI) was calculated as described in the statistical methods in the medical research book [14].

### Statistical analysis

The incidence rate and age-adjusted standardized incidence rate of hip fracture were estimated and presented in per 100,000 populations according to age, sex, and Qataris and non-Qataris of the target populations. Specific incidence rates were presented along with 95% confidence interval (CI) to measure the precision of point estimate value. Descriptive statistics were used to summarize demographic and parameters related to hip fractures. The normally distributed data and results were reported as the mean and standard deviation (SD), and the remaining results were reported as median and interquartile range (IQR). Categorical data were summarized using frequencies and percentages. All statistical analyses were performed using the statistical packages SPSS 27.0 (IBM Inc.; Armonk, NY, USA) and Epi-info (Centers for Disease Control and Prevention, Atlanta, GA) software.

### Ethical approval

This study was approved by the Medical Research Center of HMC under protocol number 01–21-489, and waiver consent was used to extract data from the subjects’ medical records.

## Results

### Demographic and hip fracture event characteristics

Over the study period, a total of 458 hip fracture events were identified; among which, 383 (83.6%) were low-energy trauma hip fractures, and 75 (16.4%) were high-energy trauma hip fracture events. Nine pathological hip fracture events were identified among the low-energy trauma hip fractures. The total number of osteoporotic hip fracture events was 374 (81.7%). The median age of the patients (IQR 25^th^–75^th^) was 69 years (range, 56–78 years), with a slight male predominance (215, 57.5%). Table 1 shows the demographic characteristics and details of the identified hip fracture events during the study period (2017–2019).

**Table 1:** Demographic characteristics of hip fracture events over the study period 2017–2019

**Variables**	
Median (IQR 25^th^–75^th^) age, years	69 (56–78)
Sex, males *n* (%)	215 (57.5%)
¶Qataris, *n* (%)	141 (37.7%)
¶Expatriates, *n* (%)	233 (62.3%)
Total number of hip fracture events, *n*	458
Low-energy hip fracture events*, *n* (%)	383 (83.6%)
High-energy hip fracture events, *n* (%)	75 (16.4%)
Pathological hip fracture events, *n* (%)	9 (2%)
Total number of osteoporotic hip fracture events, *n*	374 (81.7%)
Osteoporotic hip fracture events in 2017	112
Osteoporotic hip fracture events in 2018	126
Osteoporotic hip fracture events in 2019	136

*Low-energy hip fracture includes both osteoporotic hip fracture and pathological hip fracture. *IQR*, interquartile range

¶Number of individuals who developed osteoporotic hip fracture

### Age- and sex-specific incidence rate of osteoporotic hip fracture

Females had a higher incidence rate in all age groups > 50 years, except in the age group between 70 and 74 years, in which the mean incidence rate in men was higher than that in women. As expected, the highest incidence rate was observed in the ≥ 80 years age group, and the lowest was observed in the ≤ 50 years age group. Table 2 shows the age-sex-specific incidence rate of osteoporotic hip fracture per 100,000 people distributed according to Qataris vs. non-Qataris with the corresponding 95% CI.

**Table 2:** Age- and sex-specific incidence rate of osteoporotic hip fracture per 100,000 population distributed according to Qataris vs. non-Qataris over the study period 2017 to 2019 with corresponding 95% CI

Age groups (years)	Qataris, *n* (mean IR)		Non-Qataris, *n* (mean IR)		Qatar population, *n* (mean IR) 95% CI	
	Male	Female	Male	Female	Male	Female
40–44	2 (9.1)	2 (8.0)	7 (1.2)	1 (0.8)	9 (1.5) 0.7803–2.819	3 (1.9) 0.652–5.634
45–49	1 (5.0)	0 (0)	12 (2.9)	2 (2.4)	13 (3.0) 1.773–5.192	2 (1.9) 0.526–6.991
50–54	3 (18.0)	3 (15.2)	10 (4.0)	5 (9.9)	13 (4.9) 2.854–8.354	8 (11.4) 5.971–22.55
55–59	2 (14.7)	7 (42.1)	12 (7.5)	6 (20.4)	14 (8.1) 2.854–8.354	13 (28.2) 16.5–48.3
60–64	3 (29.8)	6 (50.0)	17 (26.1)	8 (51.4)	20 (26.6) 17.21–41.05	14 (50.8) 30.24–85.2
65–69	6 (98.2)	12 (177.5)	28 (106.6)	11 (148.2)	34 (105.0) 75.18–146.7	23 (162.2) 108.1–243.2
70–74	14 (296.3)	9 (158.8)	24 (278.3)	9 (267.0)	38 (284.7) 207.5–380.4	18 (199.1) 126.0–314.5
75–79	11 (409.1)	14 (478.1)	16 (427.0)	13 (640.3)	27 (419.6) 288.5–609.9	27 (544.5) 374.5–791.1
80 +	19 (675.1)	27 (885.8)	28 (992.8)	24 (1034.4)	47 (834.1) 627.9–1107	51 (950.0) 723.3–1247

*Qatar population includes Qataris and non-Qataris. *IR*, incidence rate; *CI*, confidence interval; *n*, number of hip fracture

Qatari females had a higher incidence rate of osteoporotic hip fracture in all age groups (< 65–69 years) than non-Qatari females. Both non-Qatari males and females had greater incidence rates in age groups ≥ 70–74 years compared to Qatari males and females.

### Age-adjusted standardized incidence rate of osteoporotic hip fracture

The annual age-adjusted standardized incidence rate of osteoporotic hip fracture per 100,000 for the population aged ≥ 50 years with the corresponding 95% CI in 2017, 2018, and 2019 was 141.7 (141.1–142.2), 140.8 (140.2–141.3), and 162.7 (162.0–163.2) for the whole Qatar population; 154.2 (153.6–154.7), 105.2 (104.7–105.7), and 176.6 (175.9–177.1) for Qataris; and 134.8 (134.3–135.4), 183.9 (183.3–184.6), and 160.4 (159.8–161.0) for non-Qataris, respectively. Table 3 demonstrates the annual age-adjusted standardized incidence rate of osteoporotic hip fracture per 100,000 individuals, with the corresponding 95% CI for the population aged ≥ 50 years.

**Table 3:** Annual age-adjusted standardized incidence rate of osteoporotic hip fracture per 100,000 people with the corresponding 95% confidence interval for population aged ≥ 50 years

Population groups	2017			2018			2019		
	Males IR (95% CI)	Females IR (95% CI)	Total IR (95% CI)	Males IR (95% CI)	Females IR (95% CI)	Total IR (95% CI)	Males IR (95% CI)	Females IR (95% CI)	Total IR (95% CI)
Qataris	143.7 (142.8–144.4)	157.0 (156.2–157.9)	154.2 (153.6–154.7)	67.0 (66.45–67.64)	139.5 (138.8–140.3)	105.2 (104.7–105.7)	145.6 (144.7–146.4)	220.0 (219.2–221.1)	176.6 (175.9–177.1)
Non-Qataris	87.2 (86.55–87.89)	213.8 (212.8–214.7)	134.8 (134.3–135.4)	169.8 (168.9–170.7)	191.0 (190.1–191.9)	183.9 (183.3–184.6)	256.8 (255.7–257.8)	195.4 (194.5–196.3)	160.4 (159.8–161.0)
Qatar population*	112.7 (111.9–113.4)	183.0 (182.1–183.8)	141.7 (141.1–142.2)	126.3 (125.6–127.1)	155.1 (154.3–155.9)	140.8 (140.2–141.3)	129.3 (128.4–130.1)	207.9 (207.0–208.8)	162.7 (162.0–163.2)

*Qatar population includes Qataris and non-Qataris. *IR*, incidence rate; *CI*, confidence interval

## Discussion

This is the first nationwide population-based study to estimate the incidence rate of osteoporotic hip fractures in Qatar. We believe that our data are representative of the Qatar population because osteoporotic hip fractures were managed mainly in hospitals operated by HMC, and fractures were captured by ICD-10 coding across all HMC facilities. Interestingly, we found no significant difference in the age-adjusted standardized incidence rate of osteoporotic hip fractures between Qataris and non-Qataris. Expatriates aged ≥ 50 years are mainly from Arab countries, with a few from South Asian countries. In 2017 and 2019, Qataris had a slightly higher incidence rate than expatriates (154 and 176 vs. 134 and 160, respectively). Our results showed that osteoporotic hip fracture rate was higher in men in the age group (70–75) which was not the case from other reports. We were not able to justify this finding.

Our figures are close to those of regional countries. Based on our data, Qataris had a standardized incidence rate ranging from 154 to 176 per 100,000 people aged ≥ 50 years, which was slightly less than Kuwaitis, who had a standardized incidence rate from 2009 to 2012, ranging between 161 and 194 per 100,000 inhabitants aged ≥ 50 years. Kuwait’s report included all hip fractures, while our data included only osteoporotic fractures [15]. In Lebanon, the age-standardized annual incidence rates (per 100,000) were 180 in men and 256 in women, which were higher than our rates. The Lebanese data included all hip fractures [16]. In general, the incidence rate of osteoporotic hip fractures in Qatar was comparable to that in regional Middle Eastern countries, and was much lower than that in North America and European countries, where the standardized incidence rate reached > 200 per 100,000 [17].

Qatar is a small geographical country with a well-established EMR network; this provided an advantage in that we were able to capture all hip fractures that occurred over the study period (2017–2019). In addition, our study is one of the few publications that included only osteoporotic hip fractures rather than all hip fractures. We believe that our results provide an accurate estimate of the incidence of osteoporotic hip fractures. We have presented our data as the age-sex-specific incidence rate and age incidence rate standardized to the world population 2010 to enable easy comparison with the figures of other countries.

There are some limitations to this study that should be addressed. First, hip fractures from private hospitals were not counted; however, most, if not all, hip fractures in Qatar were managed by the HMC as all medical services provided by HMC are accessible for Qataris and expatriates without the need for medical insurance coverage. Second, we did not calculate the 30 post-hip fracture mortality rate, which is useful for better decision-making regarding the strategy of osteoporosis prevention management at the level of the whole Qatar population.

## Conclusion

The annual age-adjusted standardized incidence rate of osteoporotic hip fracture per 100,000 inhabitants aged ≥ 50 years in Qatar was comparable to that in regional countries and much lower than that in North America and European countries.

## Data Availability

All data stored on a secured computer system and available.

## Code availability

Custom codes were used.
